# Conversion of Biosynthetic Precursors of RNA to Those of DNA by Photoredox Chemistry

**DOI:** 10.1007/s00239-014-9617-0

**Published:** 2014-04-16

**Authors:** Dougal J. Ritson, John D. Sutherland

**Affiliations:** MRC—Laboratory of Molecular Biology, Cambridge Biomedical Campus, Francis Crick Avenue, Cambridge, CB2 0QH UK

**Keywords:** Prebiotic chemistry, Photoredox, Deoxyribose, Thymine, Systems chemistry

## Abstract

Soon after the origin of RNA-based life, depletion of prebiotically synthesised ribonucleotides would have driven the evolution of a biosynthetic pathway to these key building blocks. Ribozyme-catalysed nucleosidation—the key biosynthetic step—requires that ribose and the nucleobases are produced by abiotic chemistry and are relatively stable to the conditions of their synthesis. The most plausible prebiotic synthesis of sugars involves photoreduction of cyanohydrins by hydrogen sulphide in the presence of copper(I) cyanide, and we therefore subjected ribose to these conditions whereupon it was partially converted to 2-deoxyribose. Furthermore, a derivative of uracil is reduced under similar conditions to thymine. Thus, DNA biosynthetic precursors can be formed abiotically from those of RNA allowing for an early evolutionary transition to life based on RNA and DNA.

**Electronic supplementary material:**

The online version of this article (doi:10.1007/s00239-014-9617-0) contains supplementary material, which is available to authorized users.

## Introduction

**Scheme 1:**
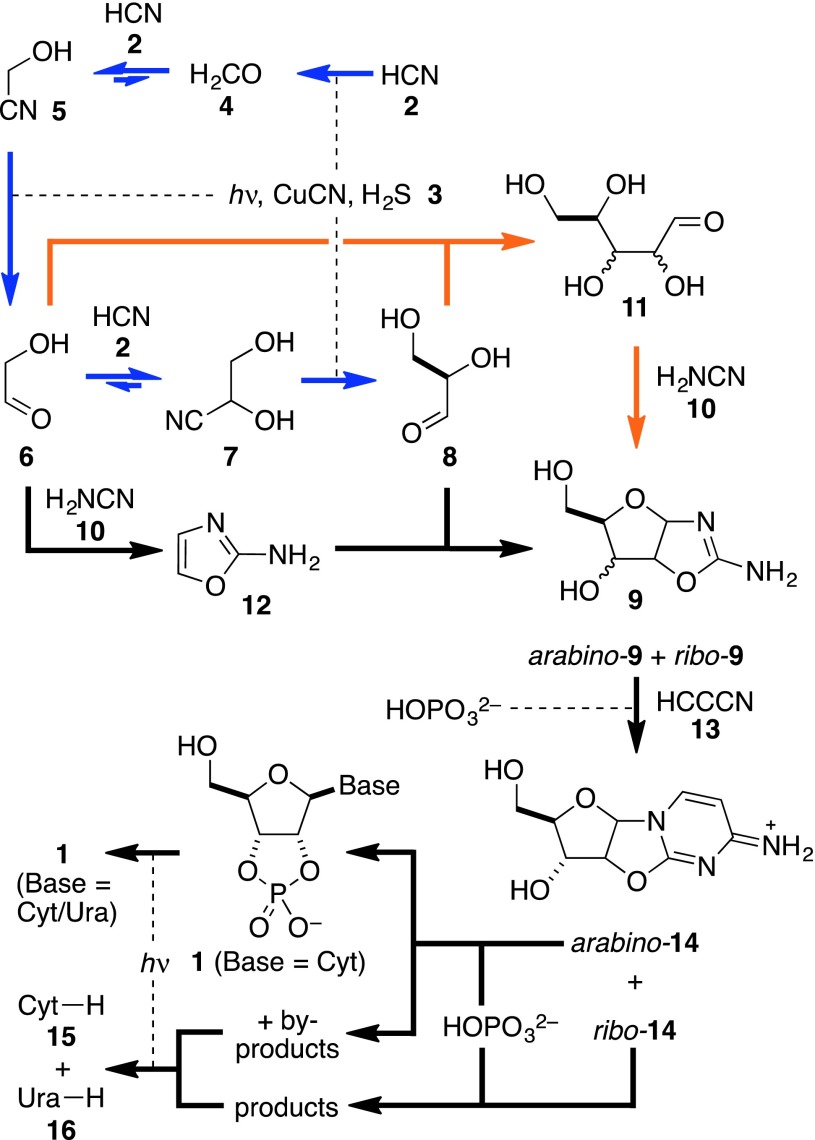
Systems chemistry synthesis of activated pyrimidine ribonucleotides

There is a substantial body of evidence that RNA played both catalytic and informational roles at the dawn of life (Joyce 2002). Furthermore, an efficient prebiotic synthesis of the pyrimidine ribonucleotides **1** (Base = Cyt/Ura) has been demonstrated (Scheme 1, black arrows) which suggests that the building blocks of RNA would have been available at the very origin of life (Powner et al. 2009; Szostak 2009; Powner and Sutherland 2010). In this synthesis, 2-aminooxazole **12** reacts with glyceraldehyde **8** to give the aminooxazolines **9**, predominantly as the *ribo*- and *arabino*-configured stereoisomers (Anastasi et al. 2006). Reaction of **9** with cyanoacetylene **13** and ensuing phosphorylation and irradiation yields **1** (Base = Cyt/Ura), with the free pyrimidine nucleobases cytosine (Cyt–H) **15** and uracil (Ura–H) **16** being major by-products of the process (Powner et al. 2009). A variation of the synthesis of **1** can be conceived (Scheme 1, orange arrows) if **6** and **8** first undergo aldol reaction giving the aldopentoses **11** (Harsch et al. 1984; Kofoed et al. 2005), which could subsequently condense with cyanamide **10** (Sanchez and Orgel 1970; Borsenberger et al. 2004; Springsteen and Joyce 2004) again yielding the aminooxazolines **9**. In either case, glycolaldehyde **6** or glyceraldehyde **8** are key, and we recently demonstrated their abiotic syntheses through the reductive homologation of hydrogen cyanide **2** (Scheme 1, blue arrows) (Ritson and Sutherland 2012; Ritson and Sutherland 2013). The reducing power for this process is provided by hydrated electrons and protons from a copper(I)⇔copper(II) photoredox cycle fuelled by hydrogen sulphide **3** (Ritson and Sutherland 2013). It is noteworthy that **2** is also the starting material for prebiotic syntheses of adenine (Ade-H) **17** and guanine (Gua-H) **18** (Sanchez et al. 1967) and these nucleobases have been converted to the corresponding ribonucleotides **1** (Base = Ade/Gua), albeit in low yield (Lohrmann and Orgel 1971; Fuller et al. 1972).

Hence, the production of ribose and the free nucleobases would appear to be an intrinsic part of the abiotic synthesis of ribonucleotides. Interestingly, ribozymes catalysing the efficient ribosylation of pyrimidine and purine nucleobases have already been isolated from random sequence RNA pools (Unrau and Bartel 1998; Chapple et al. 2003; Lau et al. 2004) suggesting the involvement of this process in early biosynthesis. However, this would require that the nucleobases **15**–**18** and ribose were stable prebiotic products. Ribose is renowned for it’s relative instability and is unlikely to have accumulated in the free form (Larralde et al. 1995) but chemically stable precursors, such as cyanohydrins **5** and **7**, could have accumulated, and then been converted to ribose abiotically in the early biotic era. This possibility would require that ribose be at least partly stable to the conditions under which **5** and **7** are reduced to glycolaldehyde **6** and glyceraldehyde **8**, respectively, and we thus set out to investigate this.

## Materials and Methods

### General Experimental

Reagents and solvents were bought from Sigma-Aldrich, Alfa Aesar and Santa Cruz Biotechnology and were used without further purification. Photochemical reactions were carried out using a Rayonet RPR-200 photochemical reactor chamber (Hg bulbs with a principle emission of 254 nm) in Spectrosil quartz cuvettes. A Mettler Toledo SevenEasy pH Meter S20 was used to monitor pH, and deoxygenation of solutions was achieved by sparging argon through the solution for 20 min. ^1^H and ^13^C NMR spectra were acquired using a Bruker Ultrashield 400 Plus machine (at 400.1 and 100.6 MHz, respectively). Samples consisting of H_2_O/D_2_O mixtures were analysed using HOD suppression to collect ^1^H NMR data. The notations s, d and br s represent singlet, doublet and broad signal and chemical shifts (δ) are given in ppm. Low resolution mass spectrometry was performed using an Agilent Technologies 1200 series/6130 Quadrupole LC/MS.

### Procedure for Photochemical Reduction of Ribose/Arabinose (Ribo-/Arabino-**11**) to 2-Deoxyribose **19**

H_2_O/D_2_O (2.5/0.4 mL) was degassed and NaH_2_PO_4_.2H_2_O (0.100 mmol, 16 mg), KSCN (0.030 mmol, 3 mg), ribose/arabinose (0.030 mmol, 4.5 mg) and NaSH.xH_2_O (60 %, 0.030 mmol, 3 mg) were added and the solution was adjusted to pH 7 using degassed NaOH/HCl. The mixture was then transferred to a quartz cuvette containing CuCN (ca. 0.5 mg, 20 mol%) which was sealed immediately. The cuvette was placed in the Rayonet reactor and the cooling fan was turned on (temperature inside reactor ca. 37 ^°^C). In the absence of mechanical stirring the reaction was sonicated for 10 s after 3 h of irradiation.

### Procedure for Photochemical Reduction of Uracil **16** to 5,6-Dihydrouracil **22**

H_2_O/D_2_O (2.5/0.4 mL) was degassed and NaH_2_PO_4_.2H_2_O (0.100 mmol, 16 mg), KSCN (0.030 mmol, 3 mg) and NaSH.xH_2_O (60 %, 0.100 mmol, 9 mg) were added and the solution was adjusted to pH 7 using degassed NaOH/HCl. Uracil (0.030 mmol, 3.3 mg) was then added and the tube was sealed and agitated until the uracil had dissolved. The mixture was transferred to a quartz cuvette which was then sealed immediately. The cuvette was placed in the Rayonet reactor and the cooling fan was turned on (temperature inside reactor ca. 37 ^°^C). The mixture was irradiated for the desired amount of time at which point an aliquot of the reaction mixture was removed and examined by NMR spectrometry.

### Procedure for Photochemical Reduction of 5-Mercaptomethyluracil **21** to Thymine **24**

H_2_O/D_2_O (2.5/0.4 mL) was degassed then 15 % (0.44 mL) of the solution was removed. NaH_2_PO_4_.2H_2_O (0.200 mmol, 32 mg), KSCN (0.030 mmol, 3 mg) and NaSH.xH_2_O (60 %, 0.030 mmol, 3 mg) were added and the solution was adjusted to pH 6 using degassed NaOH/HCl. Degassed formamide (0.44 mL) and 5-mercaptomethyluracil **21** (0.030 mmol, 4.7 mg) were added and the tube was sealed. The tube was agitated/sonicated until the solid had dissolved and the resultant solution was transferred to a quartz cuvette which was then sealed immediately. The cuvette was placed in the Rayonet reactor and the cooling fan was turned on (temperature inside reactor ca. 37 ^°^C). In the absence of mechanical stirring the reaction was sonicated for 10 s after 2 h of irradiation. The mixture was irradiated for the desired amount of time at which point an aliquot of the reaction was removed and examined by NMR spectrometry.

## Results and Discussion

The system we employed for the reduction of **5** and **7** was made by mixing a phosphate-buffered solution of the cyanohydrin and hydrogen sulphide **3** with solid catalytic copper(I) cyanide (Ritson and Sutherland 2013). In addition, it appeared that thiocyanate made the copper catalyst a more competent reductant, and so we examined it’s effect here also. Optimal reduction of the cyanohydrins to the aldehydic products **6** and **8** was observed after 2–6 h of UV irradiation of the system. We thus investigated the stability of ribose in the same system and time frame.

**Fig. 1:**
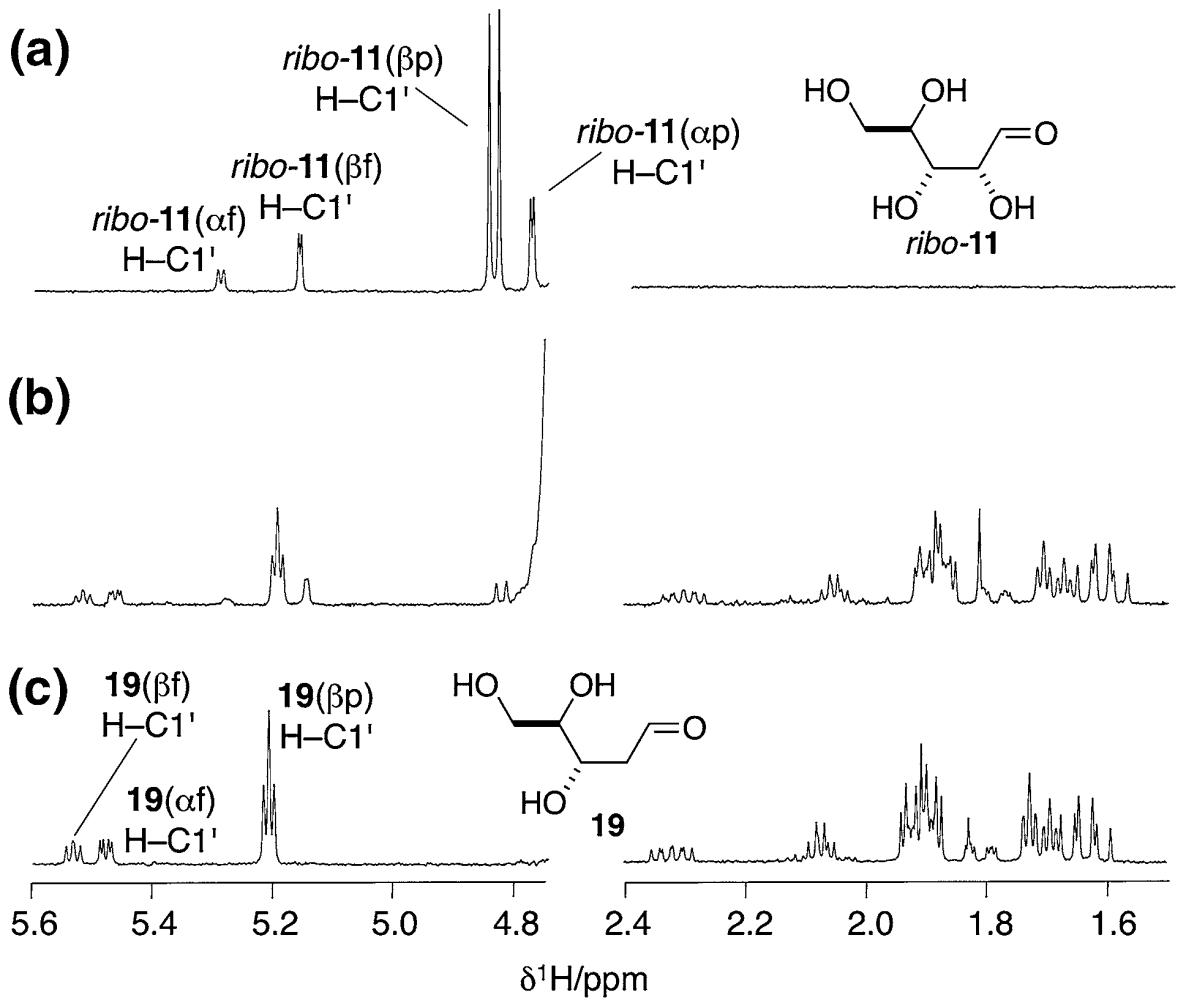
Partial ^1^H NMR analysis of the effect of exposing ribose (*ribo*-**11**) to the photoreduction conditions that generate the C_2_ and C_3_ sugars **6** and **8** from their cyanohydrin precursors **5** and **7**. **a** Spectrum of ribose in D_2_O. **b** Spectrum of the photoreduction products of ribose in H_2_O-D_2_O (with HOD signal suppression). **c** Spectrum of 2-deoxyribose **19** in D_2_O. Full ^1^H NMR spectra are shown in Supplementary Figs. S1a, b. The various tautomers of *ribo*-**11** and **19** are indicated in parentheses: α and β refer to anomeric hydroxyl group stereochemistry; f and p refer to furanose and pyranose forms

**Scheme 2:**
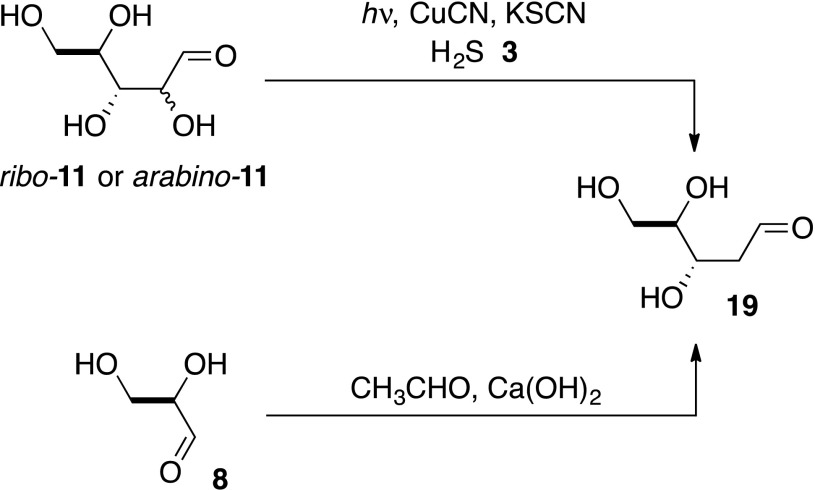
Prebiotic syntheses of 2-deoxyribose **19**

Monitoring the reaction by ^1^H NMR spectroscopy (Fig. 1) revealed that ribose was not fully stable to the reducing conditions, and after 6 h partial conversion to another sugar product or products was immediately suggested by three new anomeric proton signals downfield of the HOD signal (Fig. 1a, b). That the product or products were the result of reduction was suggested by the appearance of new signals which were upfield from any due to ribose. Suspecting that ribose might have been reduced to 2-deoxyribose **19** (Scheme 2), we compared the spectrum of the reaction products (Fig. 1b) with a spectrum of a commercial sample of **19** (Fig. 1c), whereupon this suspicion was confirmed—the three new anomeric proton signals corresponded to the α- and β-furanose and β-pyranose forms of **19**. The anomeric signal for the α-pyranose form of **19** was obscured by the HOD signal both in the spectrum of the reaction products and in that of the standard. Further support for the assignment of **19** as the reaction product was provided by comparison of the midfield region of spectra (Supplementary Fig. S1a) and sample spiking with a commercial standard. Integration of signals for ribose and 2-deoxyribose **19** relative to those of an added standard enabled quantification of the conversion after 6 h of irradiation. It was found that there was 42 % residual ribose and that **19** had been produced in 52 % yield leaving only 6 % of the starting material unaccounted for presumably as minor unidentified products. Thus, a continuous reduction and aldolisation process for the abiotic formation of ribose from the cyanohydrins **5** and **7** can be envisaged, but 2-deoxyribose **19** would appear to be an inevitable by-product.

We considered that α-deoxygenation of the aldehydic form of ribose to be the likely mechanism and, therefore, that arabinose, *arabino*-**11**, another major product of the aldolisation of **6** and **8** (Harsch et al. 1984; Kofoed et al. 2005), would also be photoreduced to the same product. In the event, *arabino*-**11** was reduced to **19** in 43 % yield (Scheme 2, Supplementary Figs. S4a, b). Investigating the system further we found that CuCN and H_2_S **3** were essential for the α-deoxygenation of *ribo*-**11** or *arabino*-**11** to proceed, whereas thiocyanate could be omitted, although the efficiency of the reaction decreased by a factor of ca. 3. This is in accordance with our previous findings and suggests that thiocyanate binds to CuCN making a more efficient catalyst for the photochemical production of hydrated electrons (Ritson and Sutherland 2013). The prebiotic synthesis of **19** has been claimed before via an aldol reaction of acetaldehyde and glyceraldehyde **8** to give 2-deoxyribose **19** in about 3 % yield (Scheme 2) (Oró and Cox 1962). In contrast, aldol reaction of glycolaldehyde **6** and **8** gives ribose in 20 % yield and arabinose in 14 % yield (Kofoed et al. 2005) and we have demonstrated photoreduction of ribose and arabinose to **19** in 52 and 43 % yield, respectively.

**Scheme 3:**
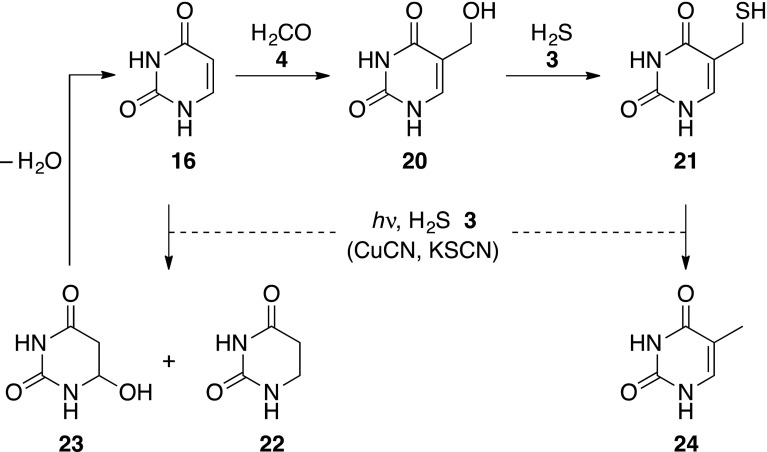
Chemistry of uracil **16** under the conditions of sugar synthesis

With a developing model for the abiogenesis of the sugars needed for early nucleic acid biosynthesis, we next turned our attention back to the nucleobases. Although **15**–**18** are intrinsically relatively stable, we recognised that they might undergo photochemical reduction or reaction with aldehyde intermediates during prebiotic ribonucleotide synthesis. In particular, we were concerned about uracil **16** as the 5,6-double bond of this nucleobase struck us as prone to saturation under the photoreduction conditions. Furthermore, **16** is known to undergo reaction with formaldehyde **4** giving rise in moderate yield to 5-hydroxymethyluracil **20** which, in turn, is converted essentially quantitatively to 5-mercaptomethyluracil **21** by reaction with hydrogen sulphide **3** (Scheme 3) (Robertson and Miller 1995).

We first investigated the stability of the nucleobases **15**–**17** towards the photoreduction conditions (guanine **18** was not investigated because it is insufficiently soluble for NMR analysis). Cytosine **15** and adenine **17** proved stable, but as we had suspected uracil **16** was not and was efficiently converted into two new products according to ^1^H NMR analysis (Supplementary Fig. S2a). One of these, dihydrouracil **22**, was indeed the product of reduction of the 5,6-double bond, and the other was the photohydrate **23** (Scheme 3) (Moore 1958). After 16 h of irradiation, 9 % of **16** remained, while **22** was produced in 62 % yield and the photohydrate **23** in 29 % yield. However, it is known that **23** undergoes elimination of water to regenerate uracil **16**, thus the partial production of **23** under the photoreduction conditions effectively protects a sizeable portion of **16** from reduction. The water elimination reaction to regenerate **16** is extremely slow at room temperature, but can be accelerated by heating (Supplementary Fig. S2b). Unlike the reduction of ribose to 2-deoxyribose, the photoreduction of **16** neither requires copper nor thiocyanate, although their presence does not affect the reduction.

We next considered the consequences of some of the prebiotically synthesised uracil **16** having reacted with formaldehyde **4**. As thiolysis of the initial hydroxymethylation product **20** to 5-mercaptomethyluracil **21** is so efficient (Robertson and Miller 1995), we reasoned that **20** would not accumulate in the presence of hydrogen sulphide **3** in the dark, but that **21** would. We then wondered what would happen if **21** was subsequently subjected to the photoreduction conditions. Although the 5,6-double bond of **21**, like that of uracil **16**, is potentially prone to saturation, the *C*–*S* bond is also a potential site of reduction. In the event, the *C*–*S* bond proved more susceptible to the reduction conditions, and the thiomethyl group of **21** was converted to a methyl group giving thymine **24** in 36 % yield after 6 h (Supplementary Figs. S3a, b). 5-Hydroxymethyluracil **20** has been reduced directly to **24** in 3 % yield by heating in 2 M formic acid (Choughuley et al. 1977), conditions which are prebiotically questionable. In contrast, the conversion of **20** to 5-mercaptomethyluracil **21** occurs by heating at neutral pH and proceeds in 99 % yield (Robertson and Miller 1995), and we have demonstrated photoreduction of **21** to **24** in 36 % yield. As with the photoreduction of uracil **16**, we found that neither copper nor thiocyanate were required for the reaction, although their inclusion in the system did not inhibit the reduction of **21** to thymine.

We have not investigated in detail the mechanisms by which the various photoreductions described herein proceed. However, we have ascertained whether all the components of the system are necessary for reduction to occur and this provides certain mechanistic clues (for a full discussion see Supplementary Table S1 and Scheme S1). Whatever the mechanisms of the reactions we have discovered, the fact that a common set of reactants and conditions converts biosynthetic precursors of RNA to those of DNA is noteworthy in the context of the earliest biology. If the ribozymes responsible for the biosynthesis of ribonucleotides had relaxed substrate specificity, they could also have been able to synthesise deoxyribonucleotides including thymidine. Furthermore, if the earliest nucleic acid polymerase also had relaxed substrate specificity, a mixed nucleic acid R/DNA, or MNA, might have been produced (Sutherland and Whitfield 1997; Trevino et al. 2011). Such a polymer offers a potential advantage over a single pure polymer in that those variants of a particular genetic sequence that is deoxyribose rich, R/DNA, would be more easily copied, whereas those which were ribose rich, R/DNA, would have been more structured, and thus more suited to catalytic roles. By polymerase gene duplication and specificity refinement, biology could evolve along a smooth trajectory from being based on R/DNA to being based on RNA plus DNA. Finally, the presence of environmental dihydrouracil **22**—from the partial reduction of uracil **16** as described herein—would have allowed its incorporation into tRNA where it contributes to essential structural flexibility (Yu et al. 2011).

In summary, it appears that RNA monomers and amino acids could have emerged simultaneously during prebiotic synthesis (Ritson and Sutherland 2013) and concurrently components of DNA would have been produced (vide supra). If a suitable abiotic coupling/activation chemistry was found, polymers of amino acids and ribonucleotides could form at a similar time and would be present before the earliest forms of life. MNA or DNA, however, would have to wait for biology to develop the requisite *N*-glycosidation ribozyme before deoxyribonucleotides could be biosynthesised.

## Electronic supplementary material

Below is the link to the electronic supplementary material.

Supplementary material 1 (PDF 8333 kb)
