# Supplementary material for: Conversion of Biosynthetic Precursors of RNA to Those of DNA by Photoredox Chemistry
Source: J Mol Evol. 2014 Apr 16;78(5):245–50. doi: 10.1007/s00239-014-9617-0 (PMC4037592; doi:10.1007/s00239-014-9617-0)
Supplement: Supplementary file 1 — Supplementary material 1 (PDF 8333 kb) [file 239_2014_9617_MOESM1_ESM.pdf]

Supporting Information for

# Conversion of Biosynthetic Precursors of RNA to those of DNA by Photoredox Chemistry

Dougal J. Ritson and John. D. Sutherland

**This PDF includes:**

Synthesis and characterisation of compounds **21** and **25**
$^{1}$H-NMR of standards **16**, **22** and **24**
Supplementary Figures S1-S4
Supplementary Table S1
Mechanistic Considerations including Supplementary Scheme S1
References

D. J. Ritson, J. D. Sutherland,
MRC - Laboratory of Molecular Biology,
Francis Crick Avenue,
Cambridge Biomedical Campus,
Cambridge, CB2 0QH, UK.
Email: johns@mrc-lmb.cam.ac.uk
Email:dritson@mrc-lmb.cam.ac.uk

**Synthesis of compounds 21 and 25**

*5-Mercaptomethyluracil* ***21***

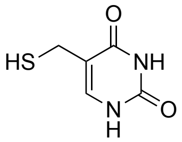


Prepared according to a literature procedure (Giner-Sorolla and Medrek 1966). Colourless solid. $^{1}$H-NMR (DMSO-d$_6$) (characterised in: Cai, Li and Taylor 2005) δ 11.10 (1 H, br s), 10.72 (1 H, br s), 7.38 (1 H, s), 3.25 (2 H, s), 2.58 (1 H, br s); $^{13}$C-NMR (DMSO-d$_6$) δ 163.5, 151.1, 138.8, 112.2, 19.9; ESI$^{+}$ *m/z* (%): 159 (MH$^{+}$, 100), 237 (12), 341 (7).

*Bis(thyminyl) disulfide* ***25*** (Giner-Sorolla and Medrek 1966)

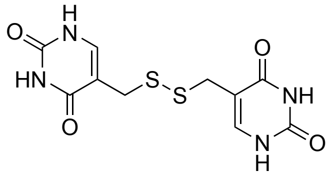


Potassium thioacetate (1.63 mmol, 187 mg) was added to a solution of 5-chloromethyluracil (250 mg, 1.56 mmol) in dry DMF (7 mL) and stirred overnight. The solution was concentrated *in vacuo*, the residue suspended in MeOH and the resultant solution concentrated (repeated twice). The crude product was suspended in water, sonicated, agitated for 10 min and the suspension then centrifuged. The supernatant was discarded and the process repeated. The residue was suspended in water, frozen and lyophilized. The crude product was used directly in the next step.

The crude thioester (50 mg, 0.25 mmol) was suspended in dry MeOH (7 mL) under air atmosphere and NaOMe (12 mg, 0.222 mmol) was added. The reaction mixture was heated to 40 °C and stirred overnight. The reaction mixture was then neutralized with HCl (pH ~ 5) and concentrated *in vacuo*. The crude product was suspended in $H_2O$, sonicated, the resultant suspension centrifuged and the supernatant discarded to give, after drying, bis(thyminyl) disulfide **25** (20 mg, 0.064 mmol, 51%) as a pale brown solid. $^{1}$H-NMR (DMSO-d$_6$) δ 11.02 (4 H, br s), 7.35 (2 H, s), 3.43 (4 H, s); $^{13}$C-NMR (DMSO-d$_6$) δ 163.3, 151.2, 140.2, 107.3, 33.3; ESI$^{-}$ *m/z* (%): 627 ([M$_2$ - H]$^{-}$, 100), 313 ([M - H]$^{-}$, 37).

# NMR Spectra of compounds 21 and 25 and standards 16, 22 and 24

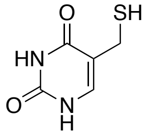


**21**

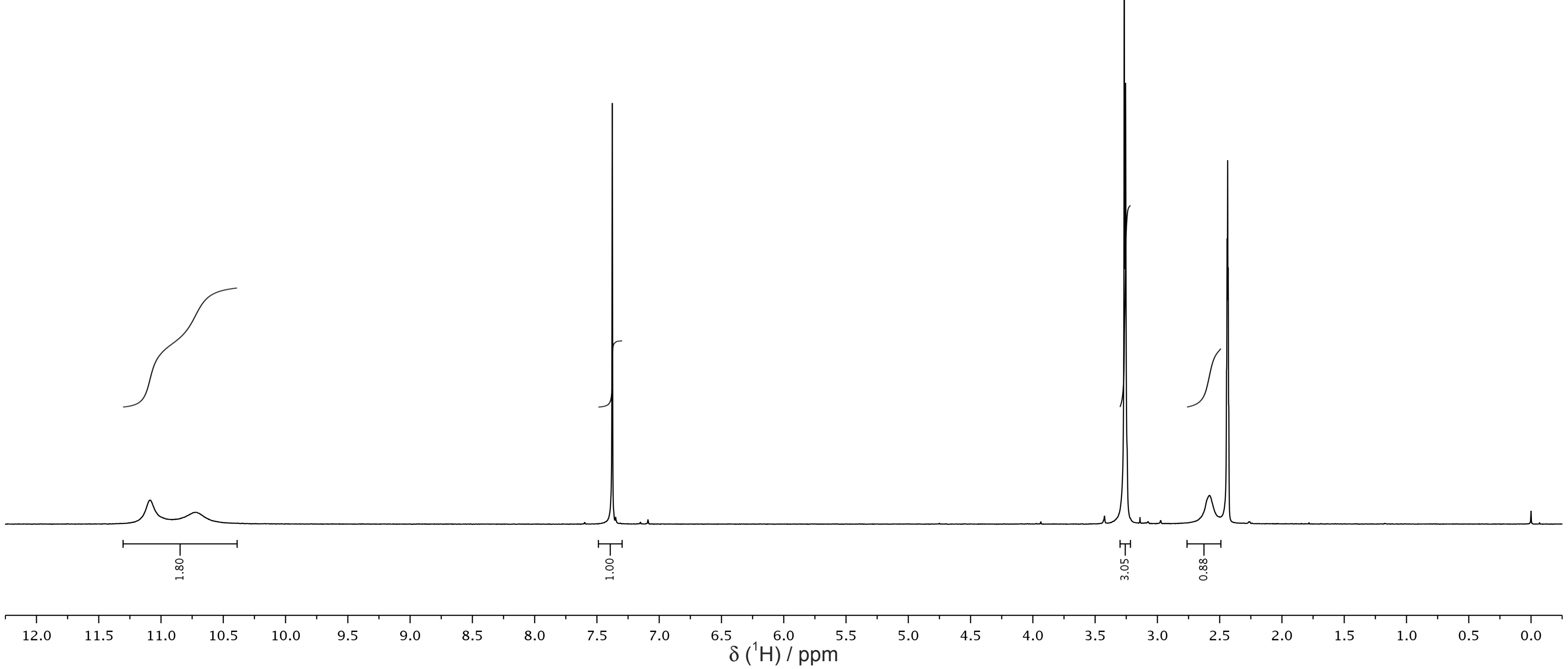

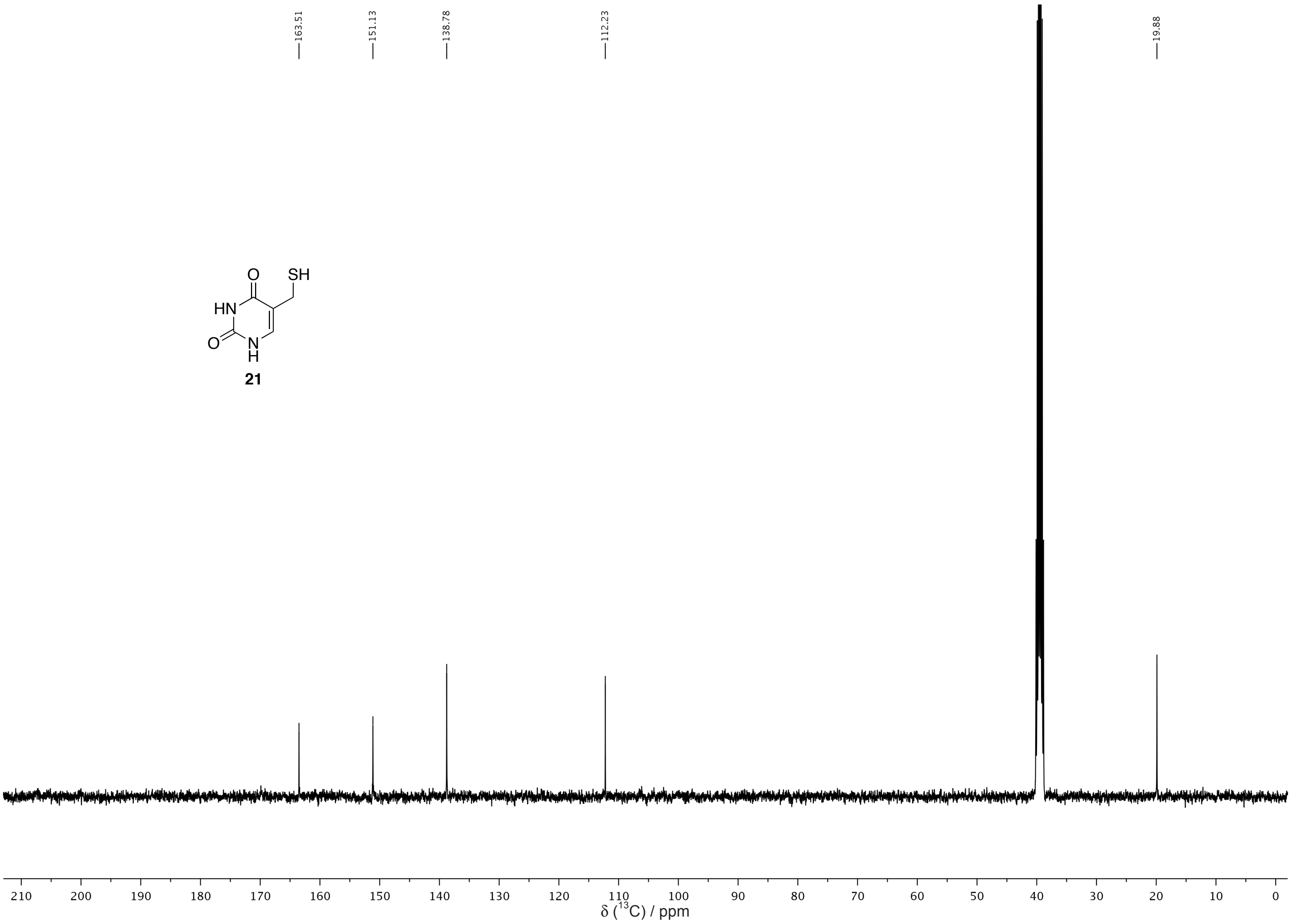
163.51
151.13
138.78
112.23
19.88
O
SH
HN
O
N
H
21
210
200
190
180
170
160
150
140
130
120
110
100
90
80
70
60
50
40
30
20
10
0
δ (13C) / ppm

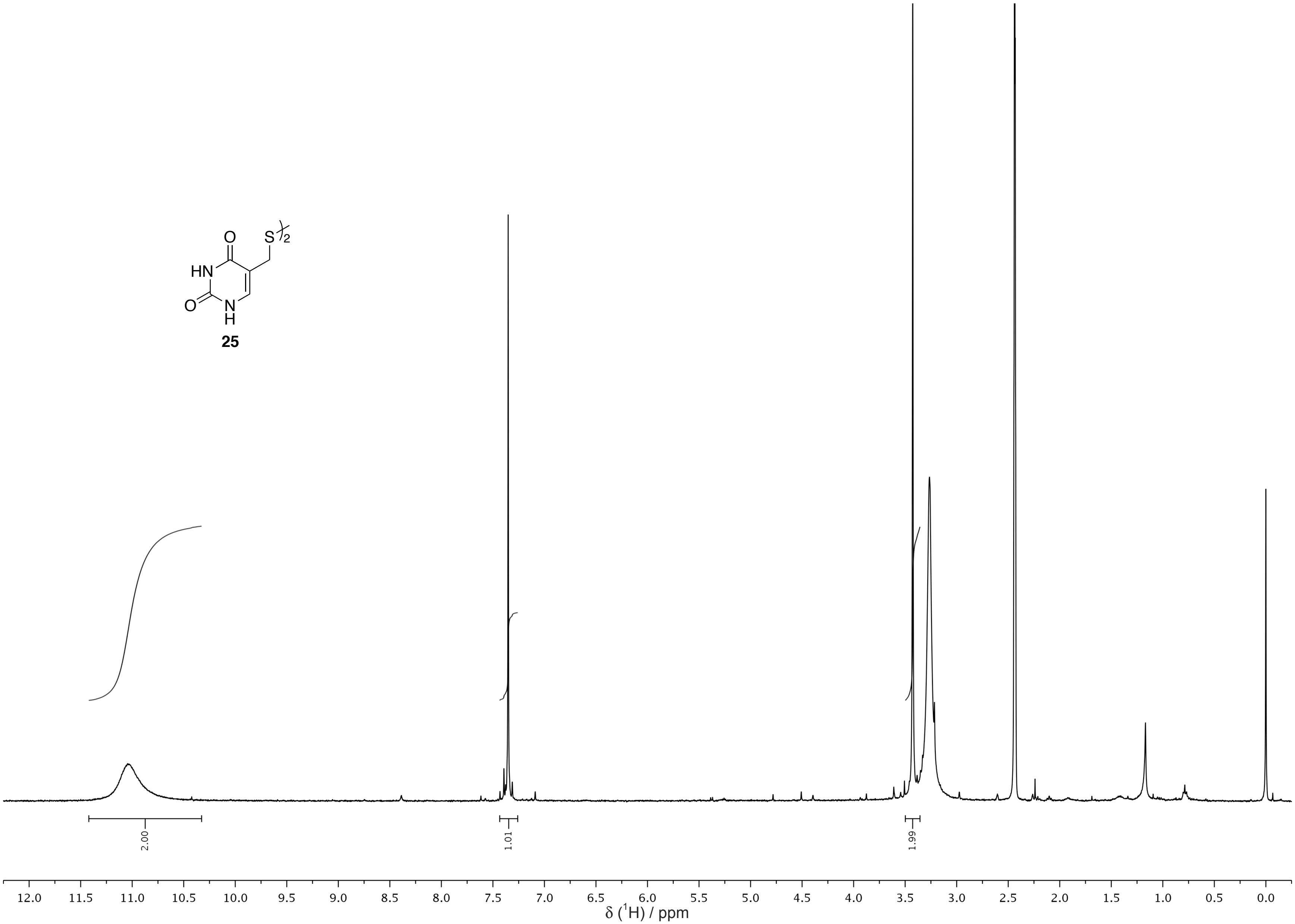
25
2.00
1.01
1.99
12.0 11.5 11.0 10.5 10.0 9.5 9.0 8.5 8.0 7.5 7.0 6.5 6.0 5.5 5.0 4.5 4.0 3.5 3.0 2.5 2.0 1.5 1.0 0.5 0.0
δ (¹H) / ppm

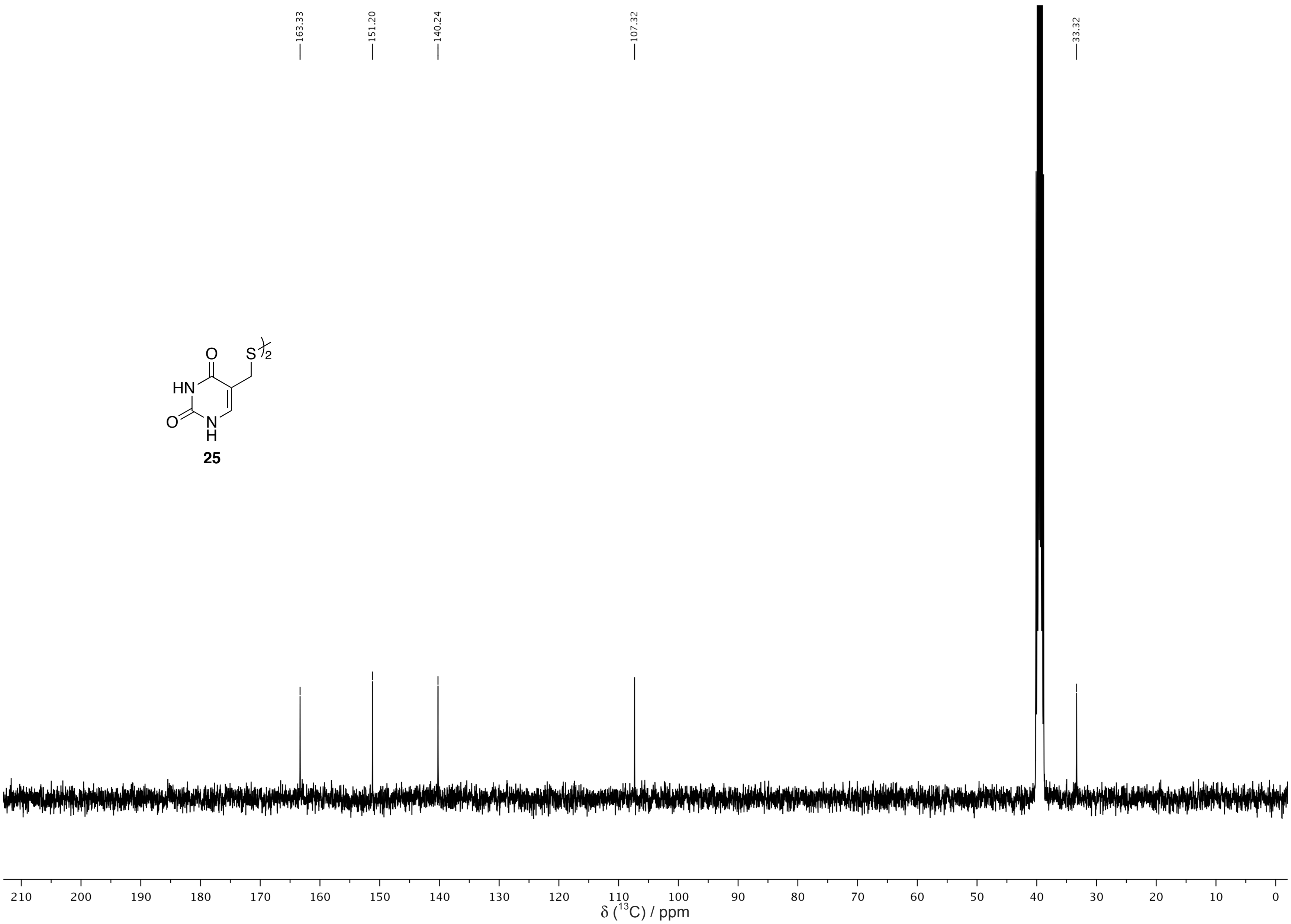
163.33
151.20
140.24
107.32
33.32
O
HN
O
N
H
S
2
25
210 200 190 180 170 160 150 140 130 120 110 100 90 80 70 60 50 40 30 20 10 0
δ ($^{13}$C) / ppm

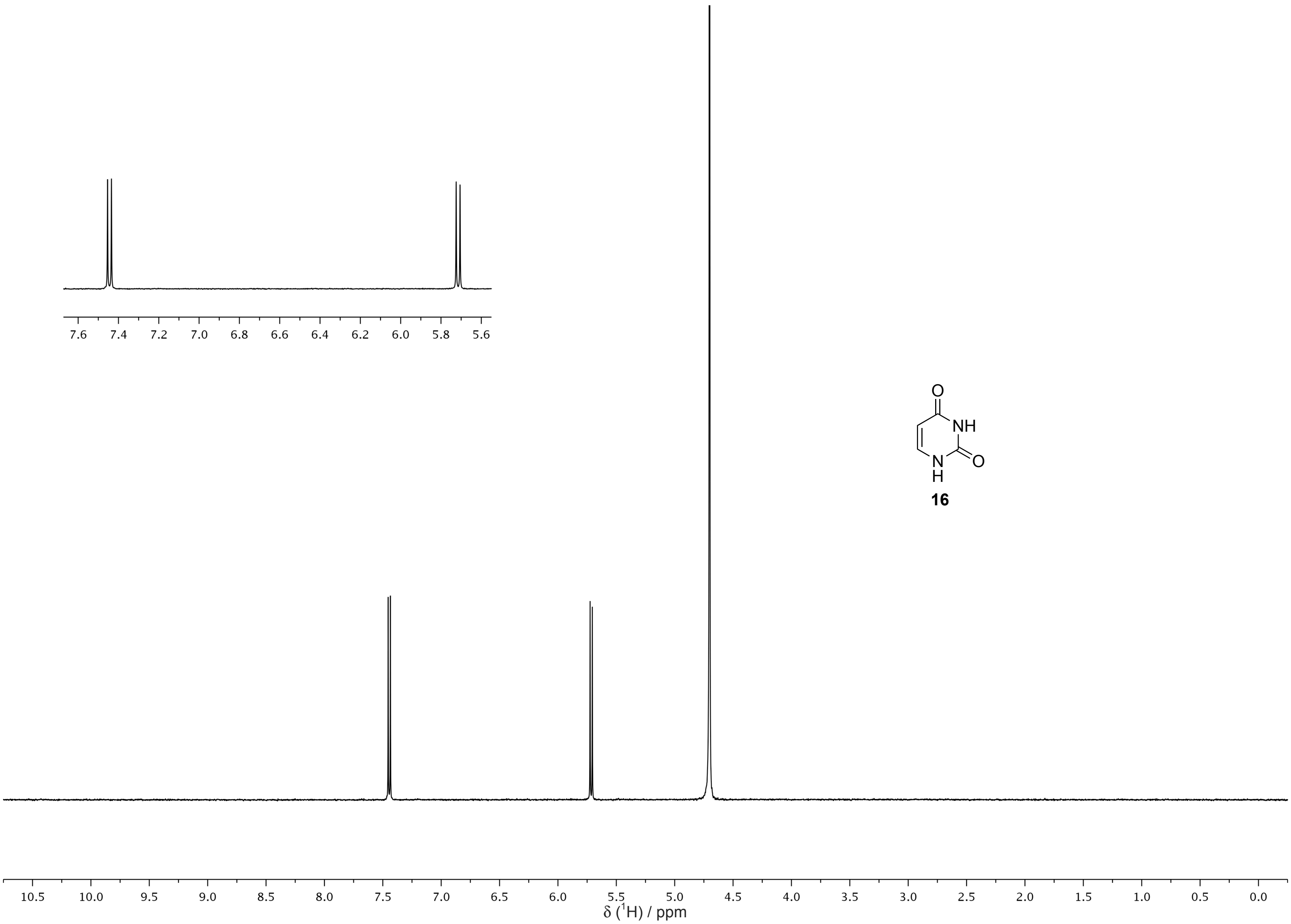
O
NH
O
N
H
16
δ (1H) / ppm

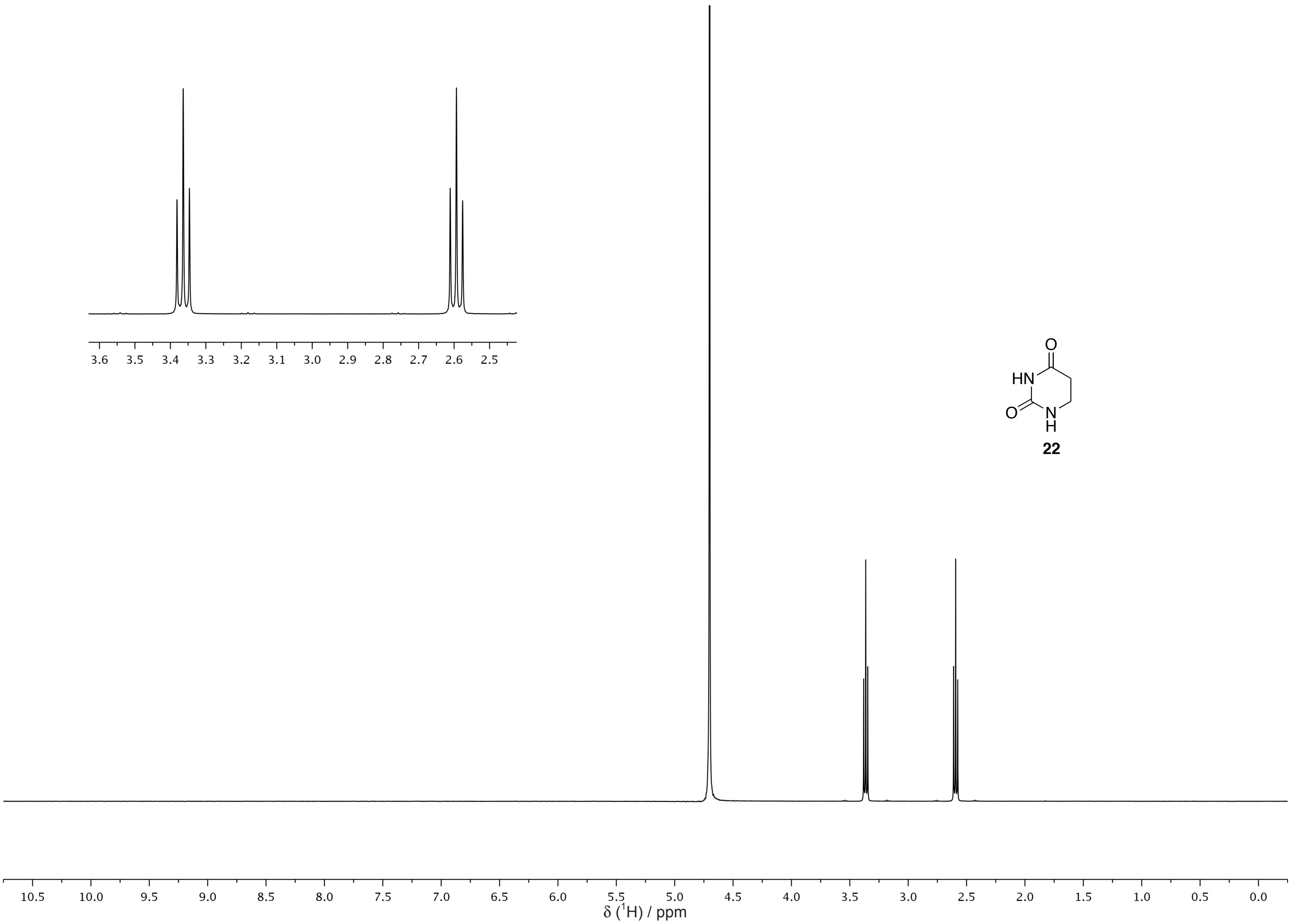
3.6 3.5 3.4 3.3 3.2 3.1 3.0 2.9 2.8 2.7 2.6 2.5
HN
O
O
N
H
22
10.5 10.0 9.5 9.0 8.5 8.0 7.5 7.0 6.5 6.0 5.5 5.0 4.5 4.0 3.5 3.0 2.5 2.0 1.5 1.0 0.5 0.0
δ (¹H) / ppm

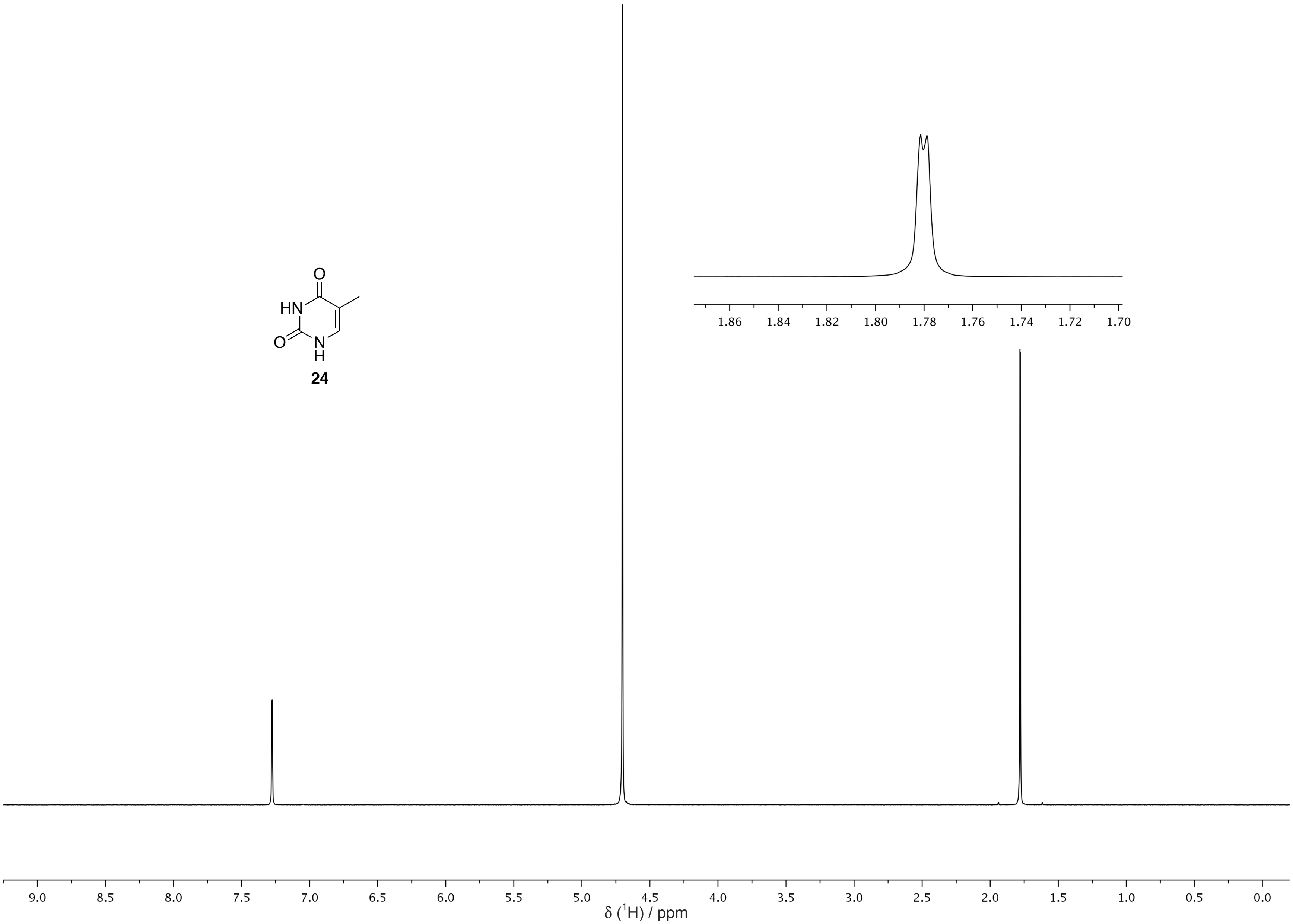

O
HN
O
N
H
24
1.86 1.84 1.82 1.80 1.78 1.76 1.74 1.72 1.70
9.0 8.5 8.0 7.5 7.0 6.5 6.0 5.5 5.0 4.5 4.0 3.5 3.0 2.5 2.0 1.5 1.0 0.5 0.0
δ ($^{1}$H) / ppm

**$^{1}$H-NMR Spectra of reactions, spiked reactions and comparisons to standards**

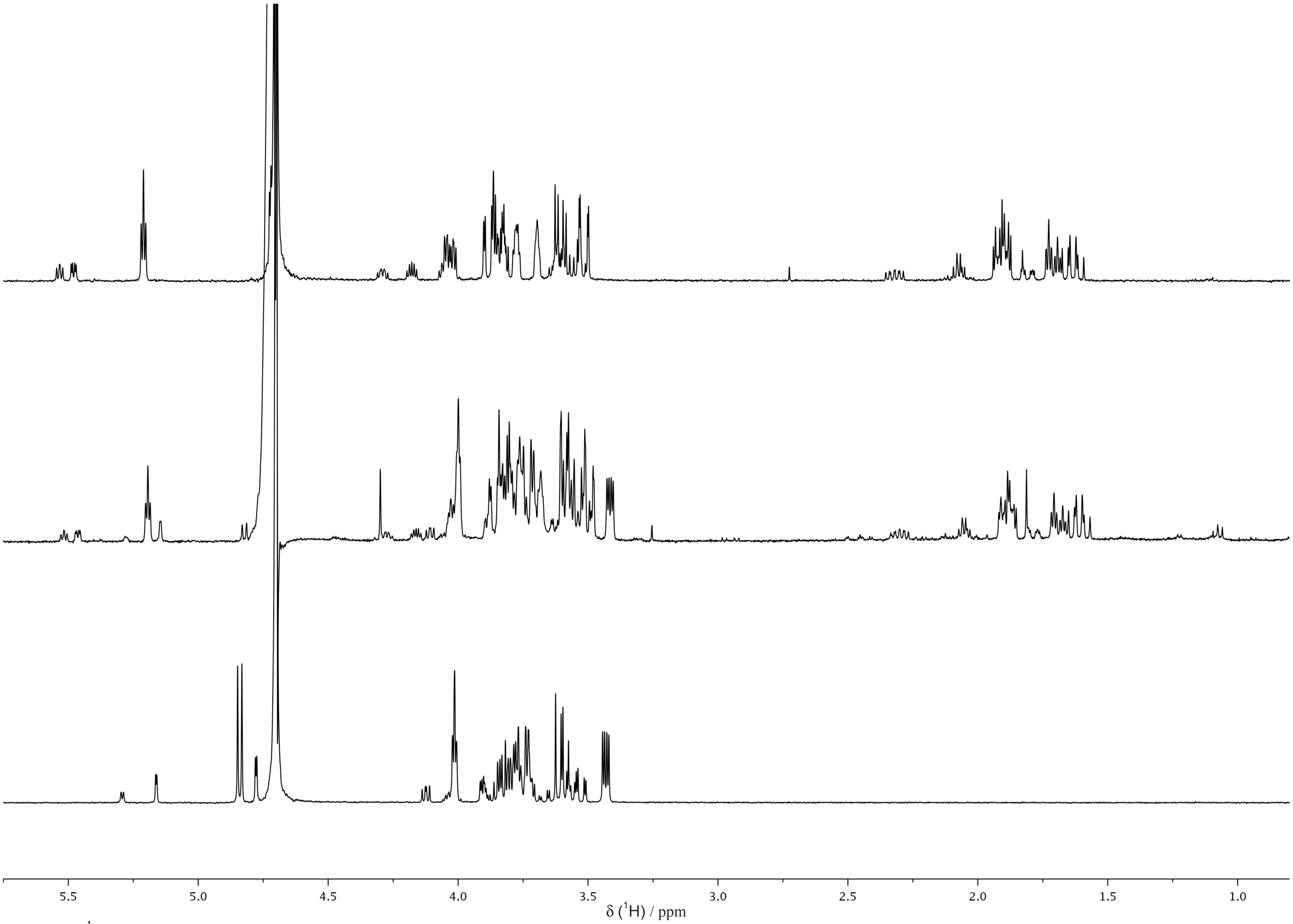


**Fig. S1a)** $^{1}$H-NMR Spectra of 2-deoxyribose **19** (upper), photoreduction products of ribose (*ribo*-**11**) (centre) and *ribo*-**11**, (lower). Singlet in centre spectrum (4.30 ppm) assumed to be glycolonitrile **5** or formaldehyde thiohydrate (Ritson and Sutherland 2013).

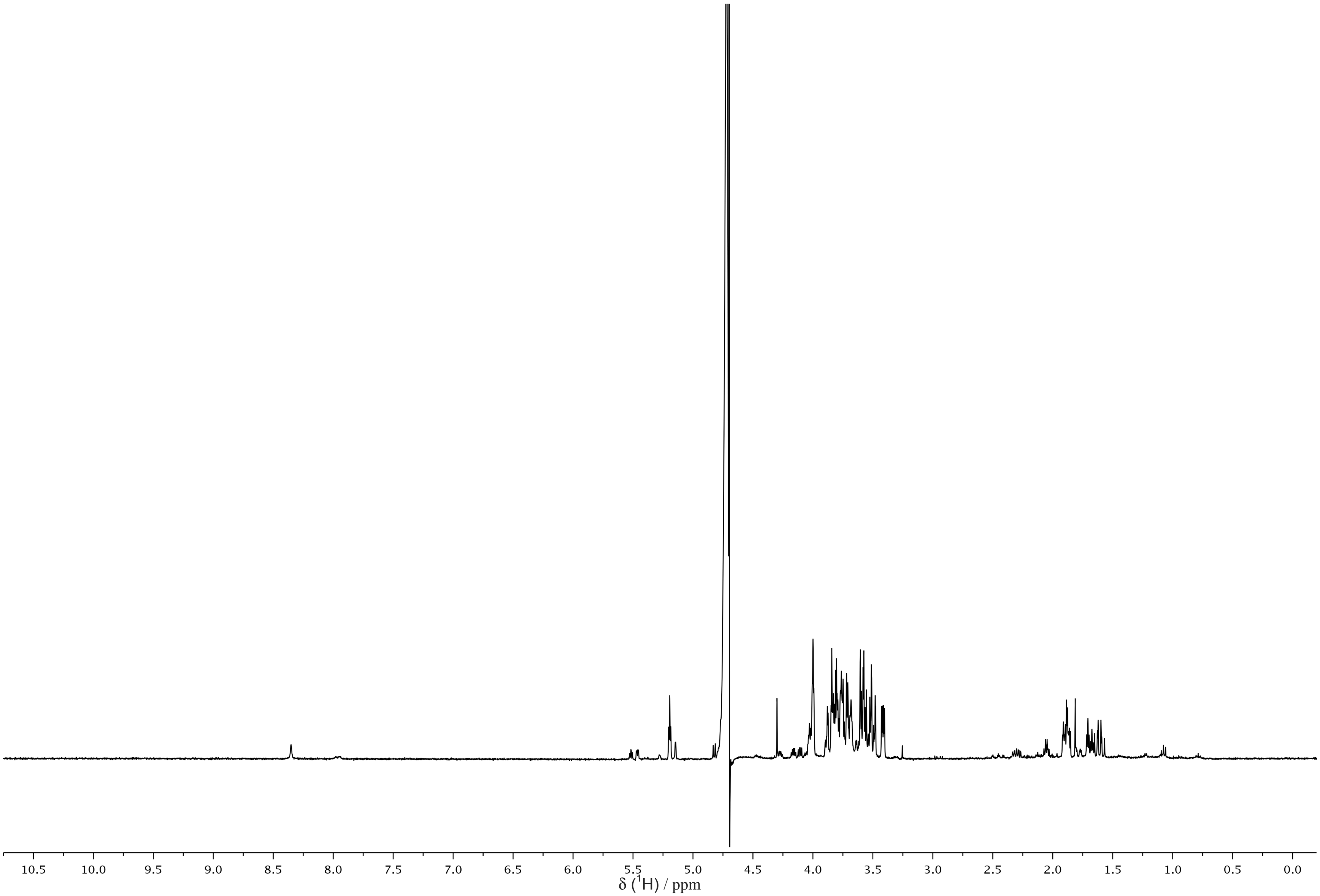


**Fig. S1b)** $^{1}$H-NMR Spectrum of the products of irradiation of ribose (*ribo*-**11**) in the presence of CuCN, NaSH, KSCN and $NaH_2PO_4$ after 6 h.

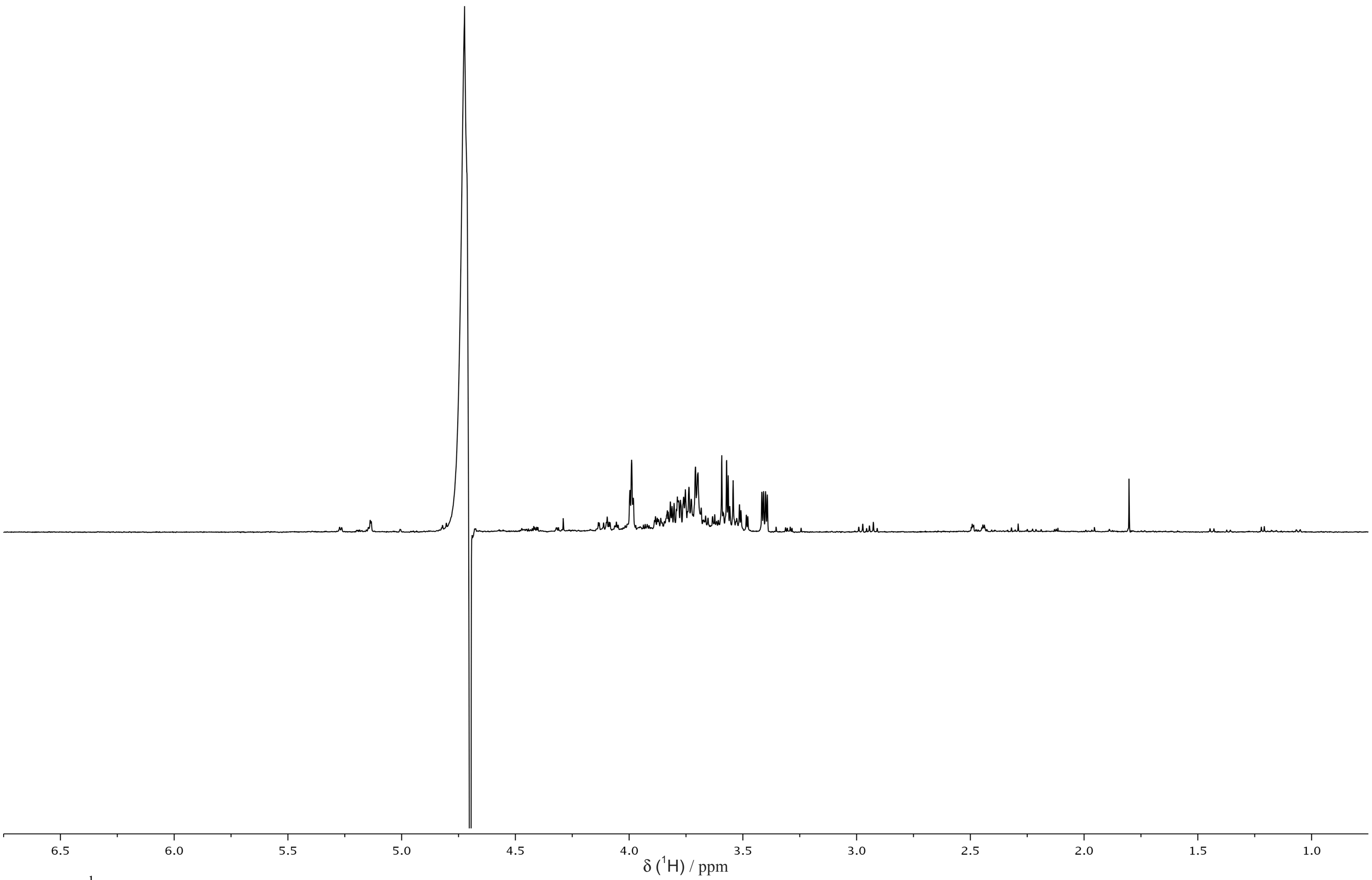


**Fig. S1c)** $^{1}$H-NMR Spectrum of the products of irradiation of ribose (*ribo*-**11**) in the presence of NaSH, KSCN and $NaH_2PO_4$ after 16 h. No 2-deoxyribose **19** is generated in the absence of CuCN.

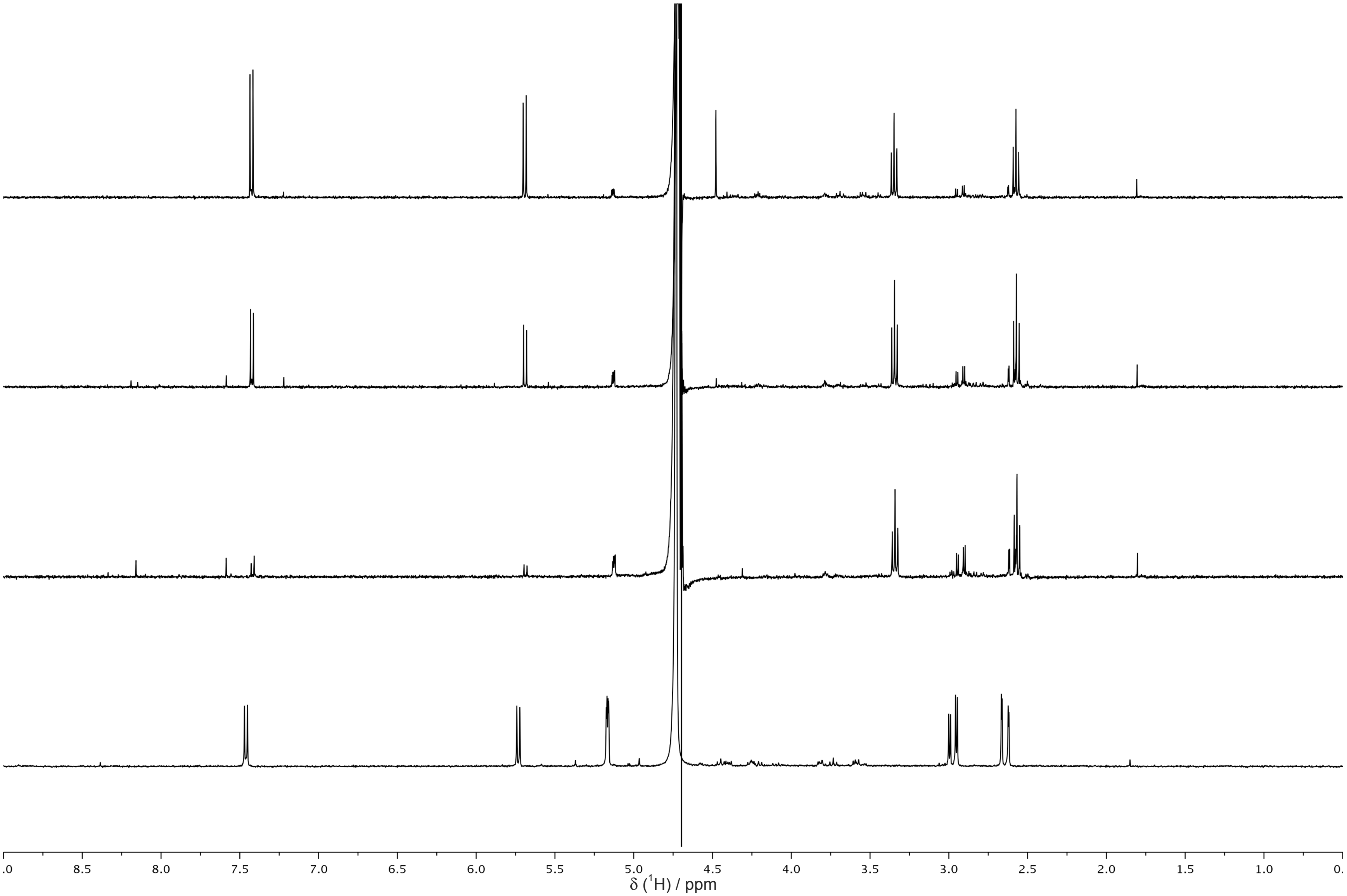


**Fig. S2a)** $^1$H-NMR Spectra of the products of irradiation of uracil **16** in the presence of NaSH, KSCN and $NaH_2PO_4$ after 4 h (upper), 8.5 h (upper centre) and 16 h (lower centre) giving 5,6-dihydrouracil **22** and uracil photohydrate **23**. Lower: $^1$H-NMR Spectrum of the products of irradiation of uracil **16** in $H_2O$ with no additives after 4 h. Uracil photohydrate **23**: $^1$H-NMR ($D_2O$) δ 5.17 (1 H, dd, *J* 4.6, 2.0), 2.97 (1 H, dd, *J* 17.4, 4.5), 2.64 (1 H, dd, *J* 17.2, 2.0).

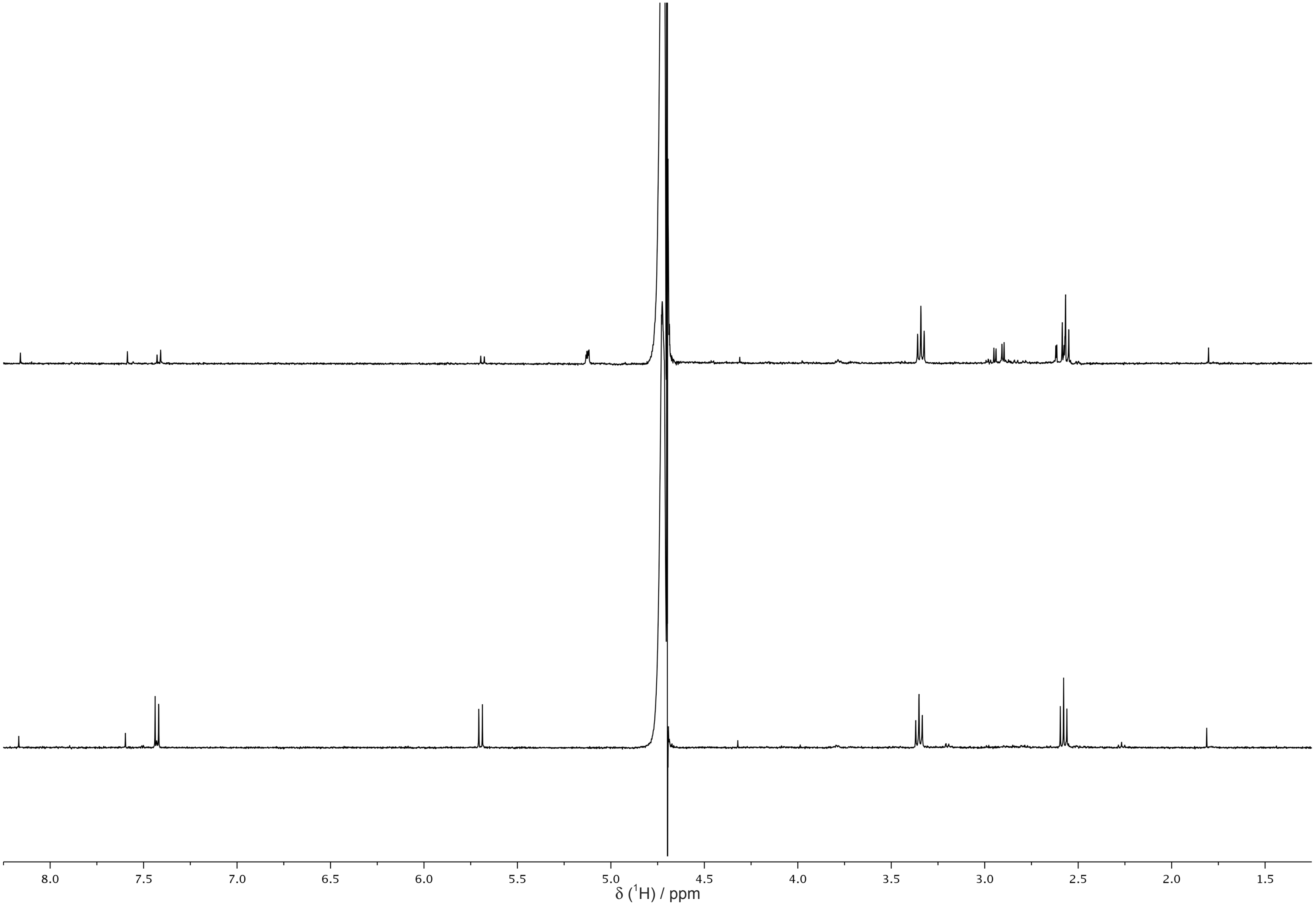


**Fig. S2b)** Reversion of uracil photohydrate **23** to uracil **16**. Upper: $^1$H-NMR Spectrum of the products of irradiation of **16** in the presence of NaSH, KSCN and $NaH_2PO_4$ after 16 h. Lower: $^1$H-NMR Spectrum of the reaction products after a subsequent period of heating (90 °C for 16 h).

| Time (h) | CuCN | Uracil **16** : | Uracil photohydrate **23** : | 5,6-Dihydrouracil **22** |
|---|---|---|---|---|
| 4 | + | 44 | 11 | 45 |
| 8.5 | + | 27 | 15 | 58 |
| 16 | + | 16 | 23 | 61 |
| 4 | – | 48 | 9 | 43 |
| 8.5 | – | 28 | 19 | 53 |
| 16 | – | 9 | 30 | 61 |

**Table S1** Products after irradiation of uracil **16**, NaSH, KSCN and $NaH_2PO_4$ at pH 7 in the presence (+) or absence (–) of catalytic CuCN (*ca.* 20 mol %). 5,6-Dihydrouracil **22** is formed at the same rate irrespective of the presence or absence of CuCN.

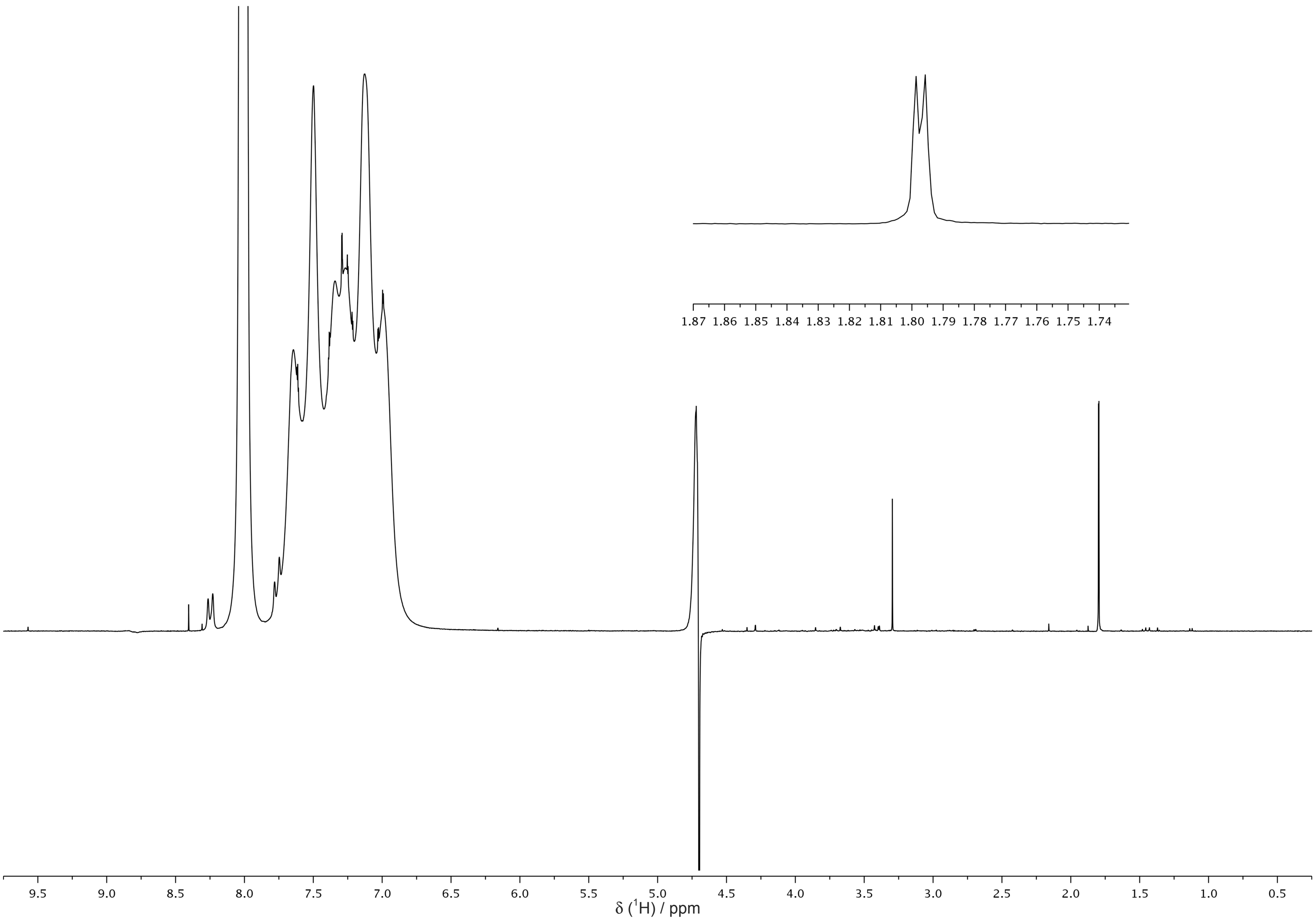


**Fig. S3a)** $^{1}$H-NMR Spectrum of the products of irradiation of 5-mercaptomethyluracil **21**, NaSH, KSCN and $NaH_2PO_4$ in $H_2O/D_2O/HCONH_2$ showing the presence of thymine **24** (singlet at δ 3.29 is due to $CH_2$ of bis(thyminyl) disulfide **25**); intense signals downfield of 6.5 ppm due to formamide. Formamide was used to solubilise **25** formed by (atmospheric) oxidation of **21**.

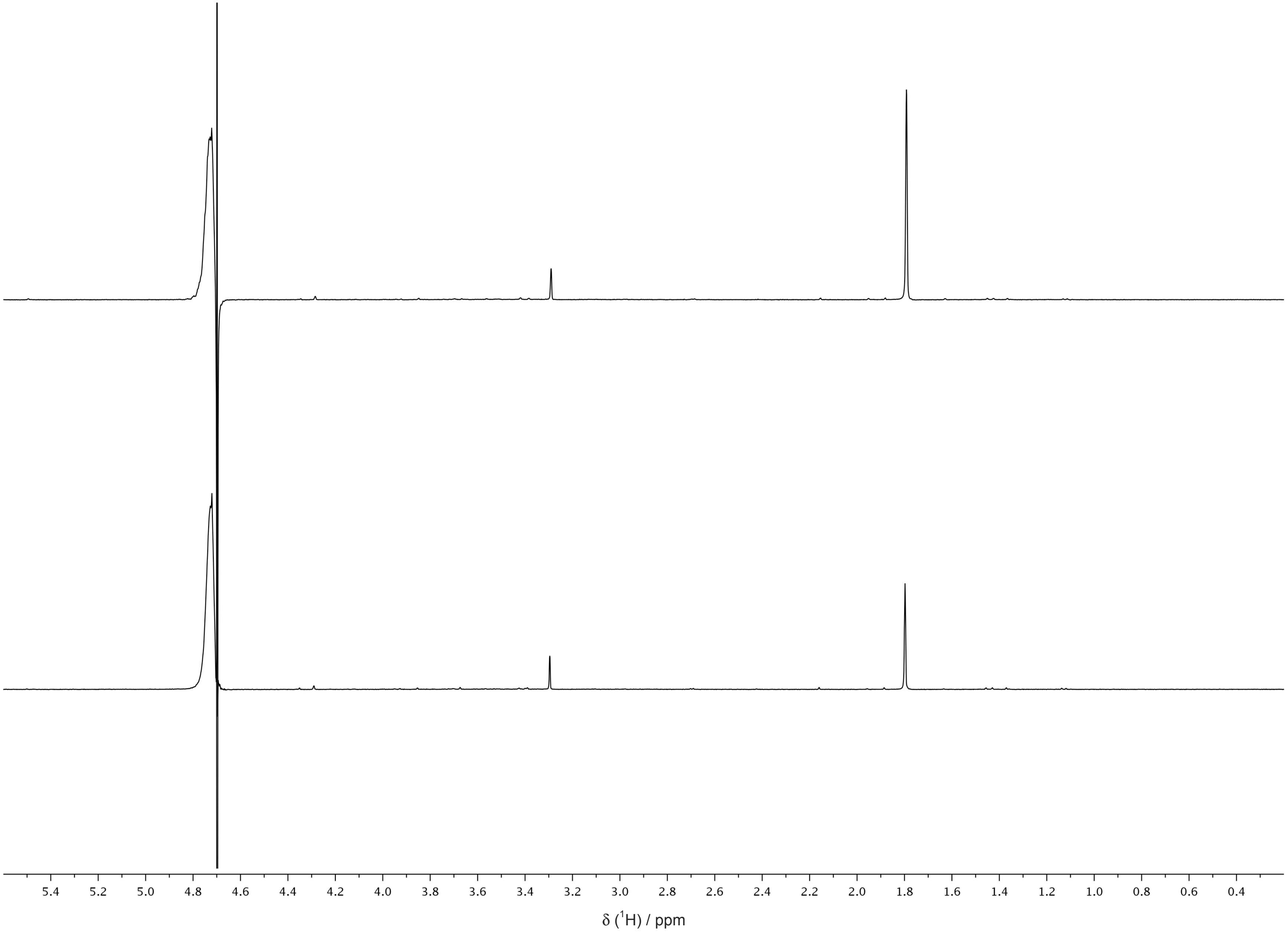


**Fig. S3b)** Lower: $^{1}$H-NMR spectrum of the products of irradiation of 5-mercaptomethyluracil **21**, NaSH, KSCN and $NaH_2PO_4$ in $H_2O/D_2O/HCONH_2$; upper: as lower spectrum, spiked with thymine **24**.

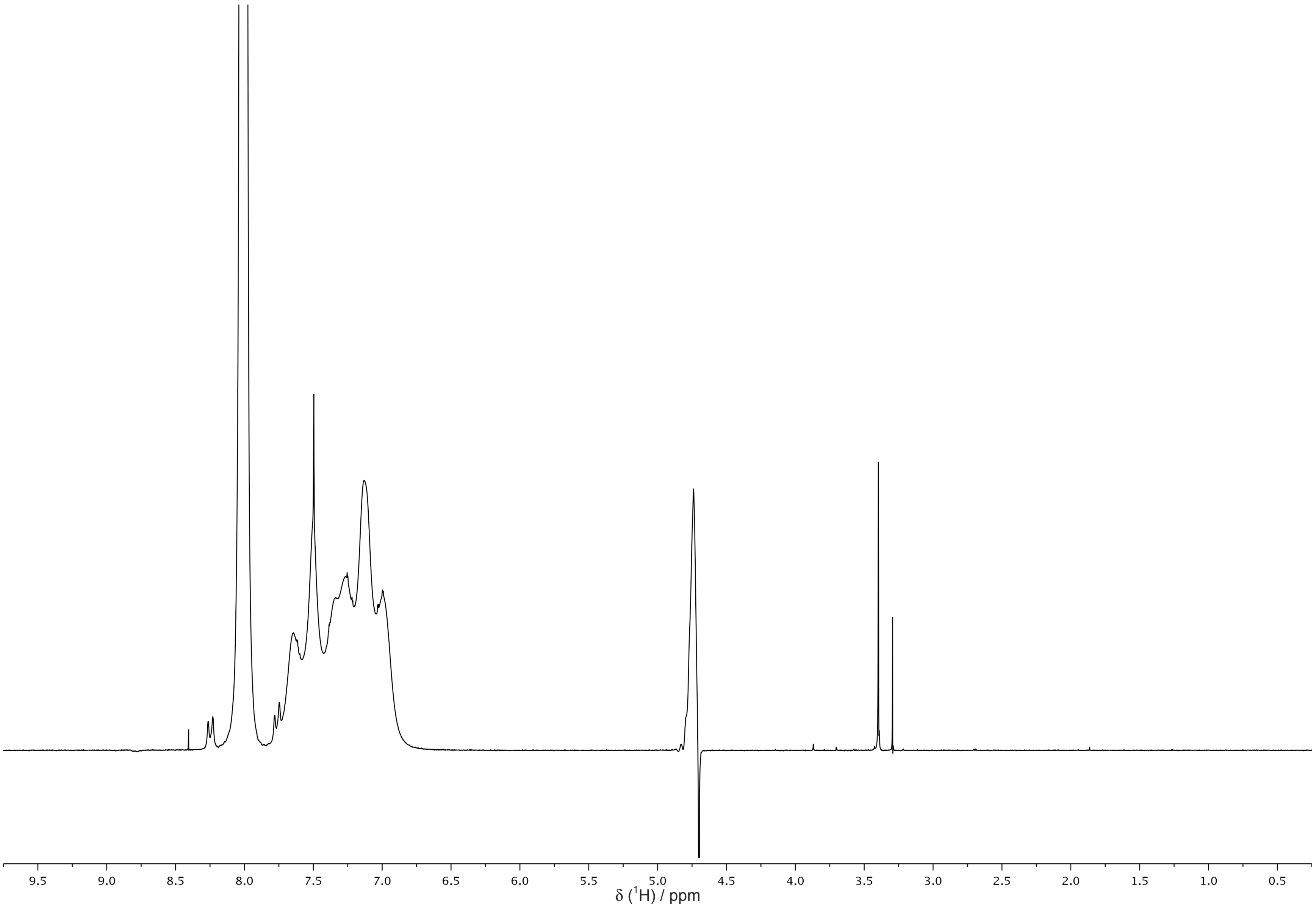


**Fig. S3c)** $^1$H-NMR Spectrum of the reaction products of 5-mercaptomethyluracil **21**, NaSH, KSCN and $NaH_2PO_4$ in $H_2O/D_2O/HCONH_2$ after 24 h in the dark. Only **21** and a small amount of bis(thyminyl) disulfide **25** are present indicating that photoexcitation is required for effective reduction.

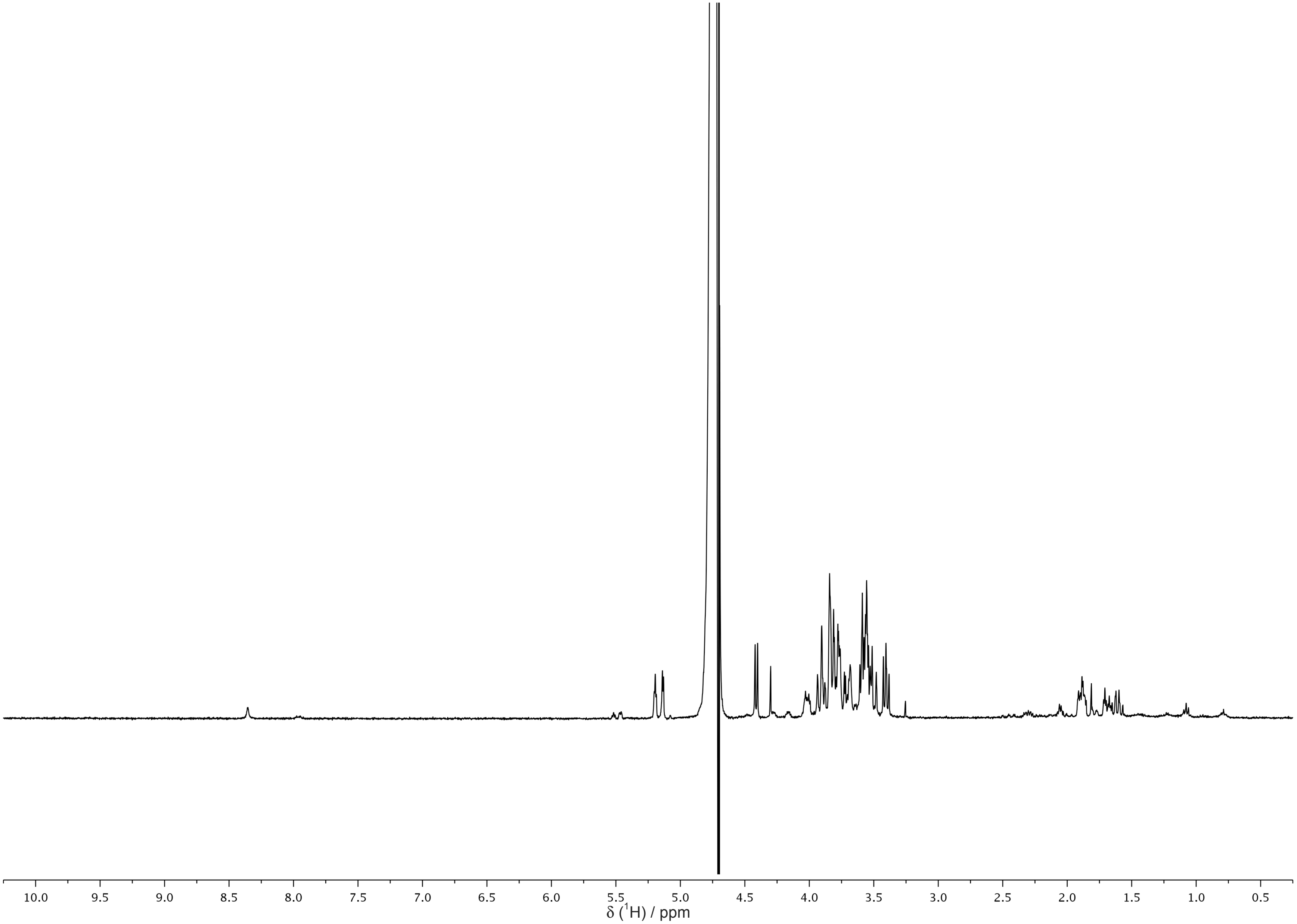


**Fig. S4a)** $^{1}$H-NMR Spectrum of the products of irradiation of arabinose (*arabino*-**11**) in the presence of CuCN, NaSH, KSCN and $NaH_2PO_4$ after 6 h.

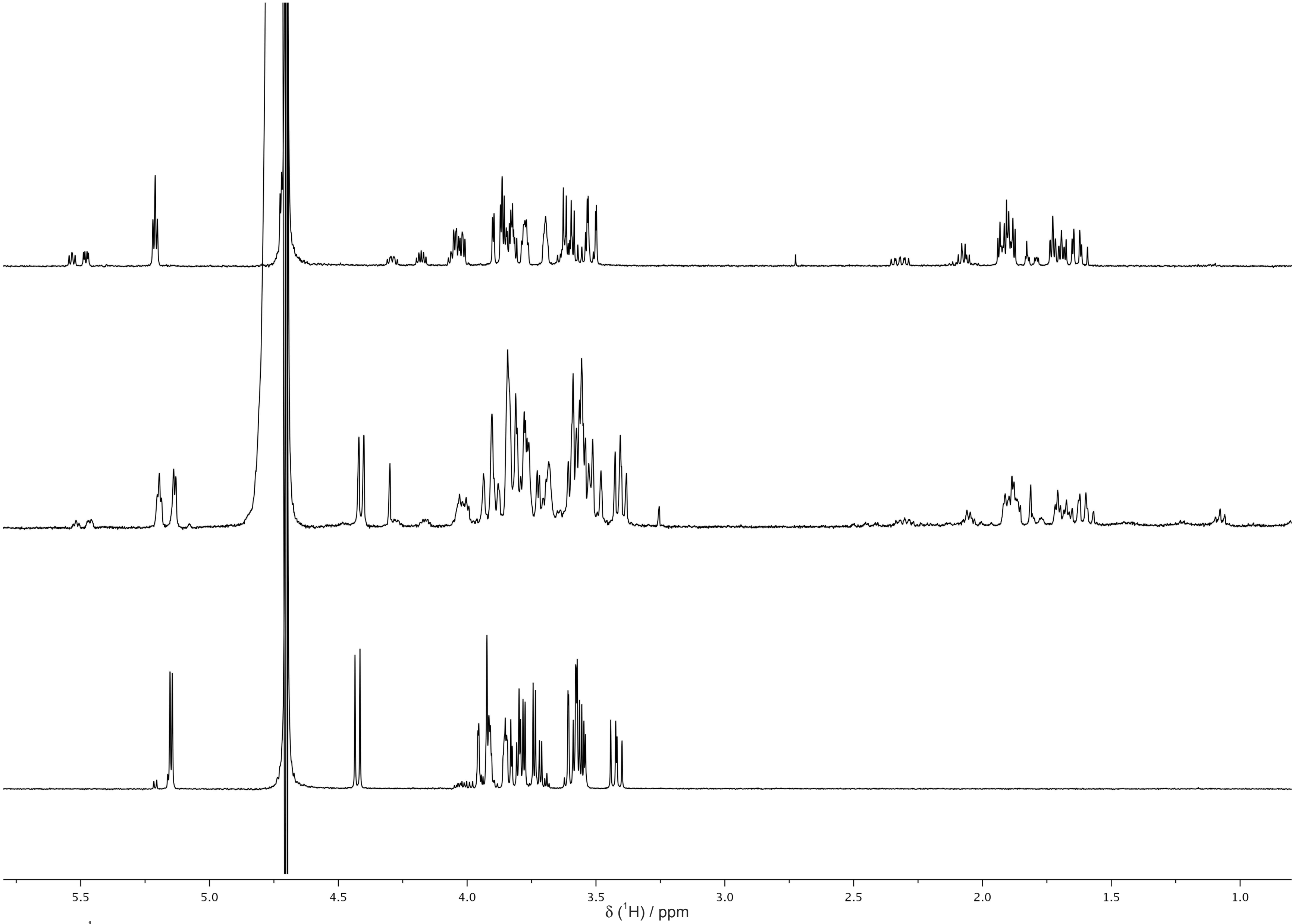


**Fig. S4b)** $^{1}$H-NMR Spectra of 2-deoxyribose **19** (upper), photoreduction products of arabinose (*arabino*-**11**) (centre) and *arabino*-**11** (lower). Singlet in centre spectrum (4.30 ppm) assumed to be glycolonitrile **5** or formaldehyde thiohydrate (Ritson and Sutherland 2013).

## Mechanistic Considerations

For the reduction of *ribo-* and *arabino-***11** to 2-deoxyribose **19** (Scheme S1a)), all the components are required and the reaction does not take place in the dark. We tentatively suggest that reduction involves (copper-assisted?) addition of a hydrated electron to the carbonyl group of **11** giving a ketyl radical anion **26**, elimination of hydroxide anion (or, through general acid catalysis by $H_2PO_4^-$, water), reduction of the resultant enolate radical **27** to an enolate anion **28**, and finally enolate protonation.

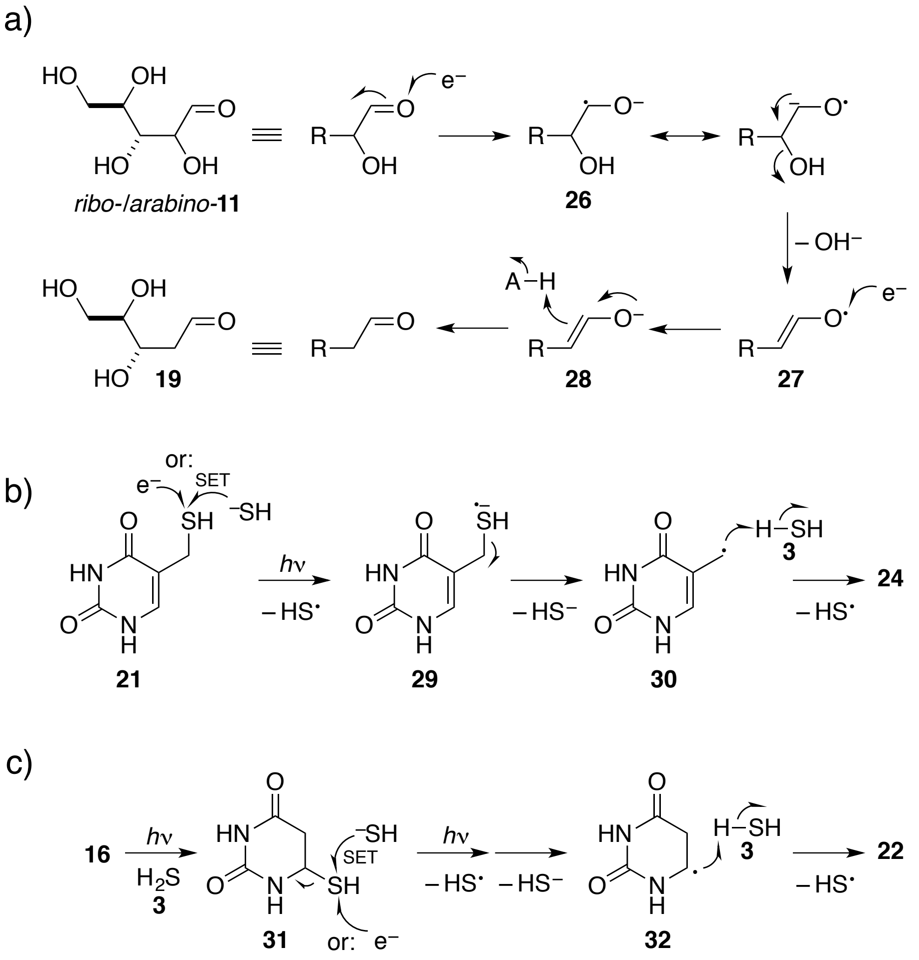


**Scheme S1** Possible photoreduction reaction mechanisms. (a) Plausible mechanism for the reduction of *ribo-* and *arabino-***11** to 2-deoxyribose **19**. (b) Mechanism for the reduction of uracil derivative **21** to thymine **24**. (c) Photoaddition of hydrogen sulfide **3** to uracil **16** followed by reduction of the resultant intermediate **31** to dihydrouracil **22**. HA is either water or $H_2PO_4^-$.

In the case of the reduction of 5-mercaptomethyluracil **21** to thymine **24**, the presence or absence of CuCN makes no difference, but the reaction did not proceed detectably in the dark (Fig. S3c)). It is known that allylic thiols are reduced extremely slowly in the dark by hydrogen sulfide

**3** by a single-electron transfer (SET) mechanism (Hebting 2003), and we therefore think that this reaction is accelerated by UV irradiation – this would involve light-promoted SET from the hydrosulfide anion $HS^-$ to the sulfur atom of **21** – or that a hydrated electron adds to the sulfur atom of **21** (Scheme S1b)). In either case, electron addition would be followed by loss of $HS^-$ from the resultant radical anion **29** giving an allylic radical **30**. Hydrogen atom transfer from **3** to this radical would then furnish **24**. The reduction of uracil **16** to dihydrouracil **22** (Scheme S1c)) also proceeds equally well regardless of whether CuCN is present or not. Whichever photoexcited state, or intermediate derived therefrom, it is that undergoes addition of water giving the photohydrate **23** (Moore 1958), it is also likely to undergo attack by hydrogen sulfide **3** giving an analogous addition product **31**. Light-promoted SET or hydrated electron addition to the sulfur atom of **31** could then be followed by loss of $HS^-$ with concommitant formation of a 5,6-dihydrouracil-6-yl radical **32**, and subsequent hydrogen atom transfer from **3** giving **22**.

## References


Giner-Sorolla A, Medrek L (1966) Synthesis and properties of 5-mercaptomethyluracil and related derivatives. J. Med. Chem. 9:97-101.

Cai J, Li X, Taylor JS (2005) Improved nucleic acid triggered probe activation through the use of a 5-thiomethyluracil peptide nucleic acid building block. Org. Lett. 7:751-754.

Hebting Y, Adam P, Albrecht P (2003) Reductive desulfurization of allylic thiols by $HS^-/H_2S$ in water gives clue to chemical reactions widespread in natural environments. Org. Lett. 5:1571-1574.
